# Research on residual strength characteristics of high water materials based on improved H-B criterion

**DOI:** 10.1371/journal.pone.0245018

**Published:** 2021-06-28

**Authors:** Lihui Zheng, Yuejin Zhou, Mingpeng Li, Xiaotong Li

**Affiliations:** 1 State Key Laboratory for Geomechanics & Deep Underground Engineering, China University of Mining & Technology, Xuzhou, China; 2 School of Mechanics and Civil Engineering, China University of Mining & Technology, Xuzhou, China; University of Science and Technology Beijing, CHINA

## Abstract

To develop a new gangue polymer filling material with low compressive ratio, this paper intends to add high water cementing material to the gangue for backfilling. Uniaxial and tri-axial bearing experiments were conducted to study its bearing characteristics and residual strength. Based on Hock-Brown model theory, it is proposed that friction angle *φ*_*r*_ can be introduced to substitute model parameter *m*_*i*_, and the degree of cohesion loss can characterize the value of s. So the improved H-B model is established to characterize the residual strength of materials with ductile failure characteristics. The results show that the compressive strength of high water filling material increases linearly corresponding to the rise of confining pressure, and its strength characteristics conform to Mohr-Coulomb strength criterion. The ductile failure characteristics of the sample endow it with high residual strength, which in turn qualifies it for underground filling. After the introduction of cohesion and friction angle, the improved H-B criterion can fit the residual strength curve of the high water filling material more competently. The fitting coefficient of the samples with three water contents is 1.00, 0.99, and 1.00, respectively. The improved H-B model of residual strength demonstrates the change rule of residual strength of the samples corresponding to the change of confining pressure; under tri-axial loading, the angle between fracture surface and axial direction becomes larger as the confining pressure rises; and the failure mode of the material transforms from splitting failure to shear failure.

## 1. Introduction

China’s energy structure is dominated by coal, and the extraction of coal causes damage to farmland and ecological environment [1–3]. Backfill mining can improve the recovery rate by achieving non-pillar mining; meanwhile it can also control the ground pressure effectively, reduce ground surface settlement, and thus realize the safe and efficient mining of coal resources [4, 5]. Currently, the filling materials in use include coal gangue, tailing sand, high water material, and paste and so on [6–8]. In recent years, high-water materials have been widely used in underground projects because of their high water content, high quality and low price. Many researchers have studied the hydration mechanism, strength and bearing characteristics of high-water materials [9–13]. The high-water material is a new kind of filling material, the physical and mechanical properties of high-water material directly affect the filling stability [14]. Solid particles and water are the main components of high-water material, the relative content of which directly affect its physical and mechanical properties. The spread of paste backfill is mainly related to mass fraction, and the effect of cement-tailings ratio on it is small [15]. The spread of paste backfill decreases with the increase in mass fraction, yield stress, and viscosity. The coarse aggregate’s specific surface area and chemical composition in the unclassified tailings-coarse aggregate paste are the main factors influencing the setting time. With the increase in the tailings-coarse aggregate ratio, the plastic viscosity of paste backfill slurry increased [16]. HOEK-BROWN proposes an empirical failure criterion for complete rocks [17]. After many developments and improvements, it has become one of the most widely used failure criteria in the field of geotechnical engineering [18–20]. Some researchers have summarized three methods of post-peak mechanical behavior of rocks, indicating that within a certain range of confining pressure, the post-peak stage of rocks will exhibit strain softening behavior and have a certain residual stress [21]. Some other researchers have summarized the methods of determining the residual strength of rocks. The residual strength values of rocks under different confining pressure conditions are determined through triaxial compression tests of rocks, and then the residual strength parameters of rocks are fitted by the M-C model or the H-B model [22]. The determination of residual strength plays an important role in rationally evaluating the stability of geotechnical engineering and effectively exerting the bearing capacity of rocks [23].

This study conducted experimental research on the stress-strain curve, bearing characteristics, and residual strength of high water-cementing material with different water contents. Based on Hock-Brown model theory, it is proposed that friction angle *φ*_*r*_ can be introduced to substitute model parameter *m*_*i*_, and the degree of cohesion loss can characterize the value of s. So the improved H-B model is established to characterize the residual strength of materials with ductile failure characteristics. The research results can provide reference and guidance for filling mining.

## 2. Experiment on the bearing characteristics of high water material

### 2.1 Source and composition of high water materials

The high-water material used in the test was taken from the Yineng Coal Mine in Shandong Province. The high-water material consists of material A and material B. Material A mainly includes sulfoaluminate cement, suspending agent and retarder, and material B mainly includes lime, Gypsum, suspension agent, quick-setting and early-strength agent. The setting time of material A and material B used alone is greater than 24h. But when the two are mixed in 1:1, it can condense in 30 minutes. The high-water material can better contact the roof to achieve the purpose of effectively controlling the surface settlement.

### 2.2 Uniaxial bearing characteristics

**Fig 1:**
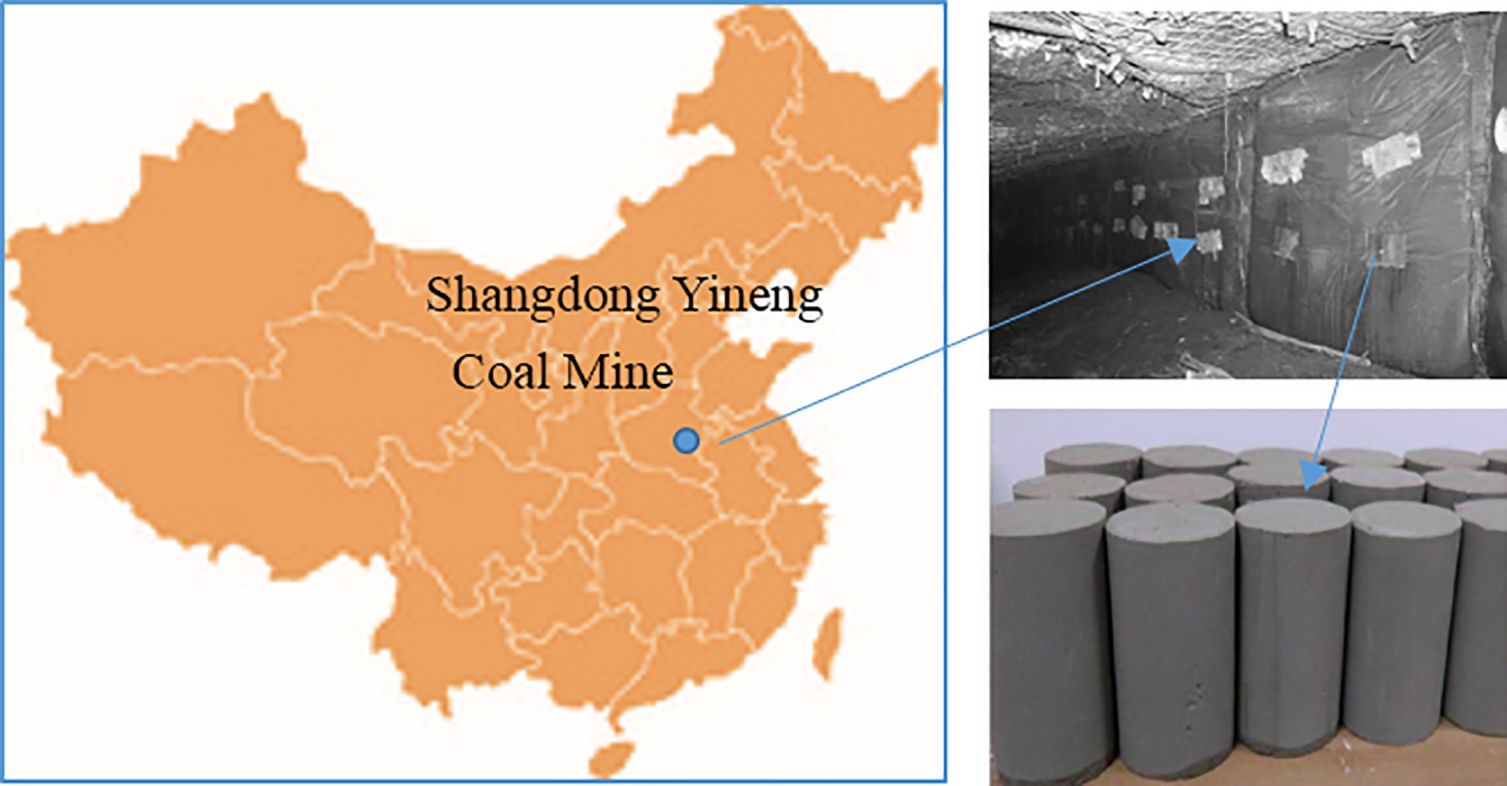
Source of high water materials.

**Fig 2:**
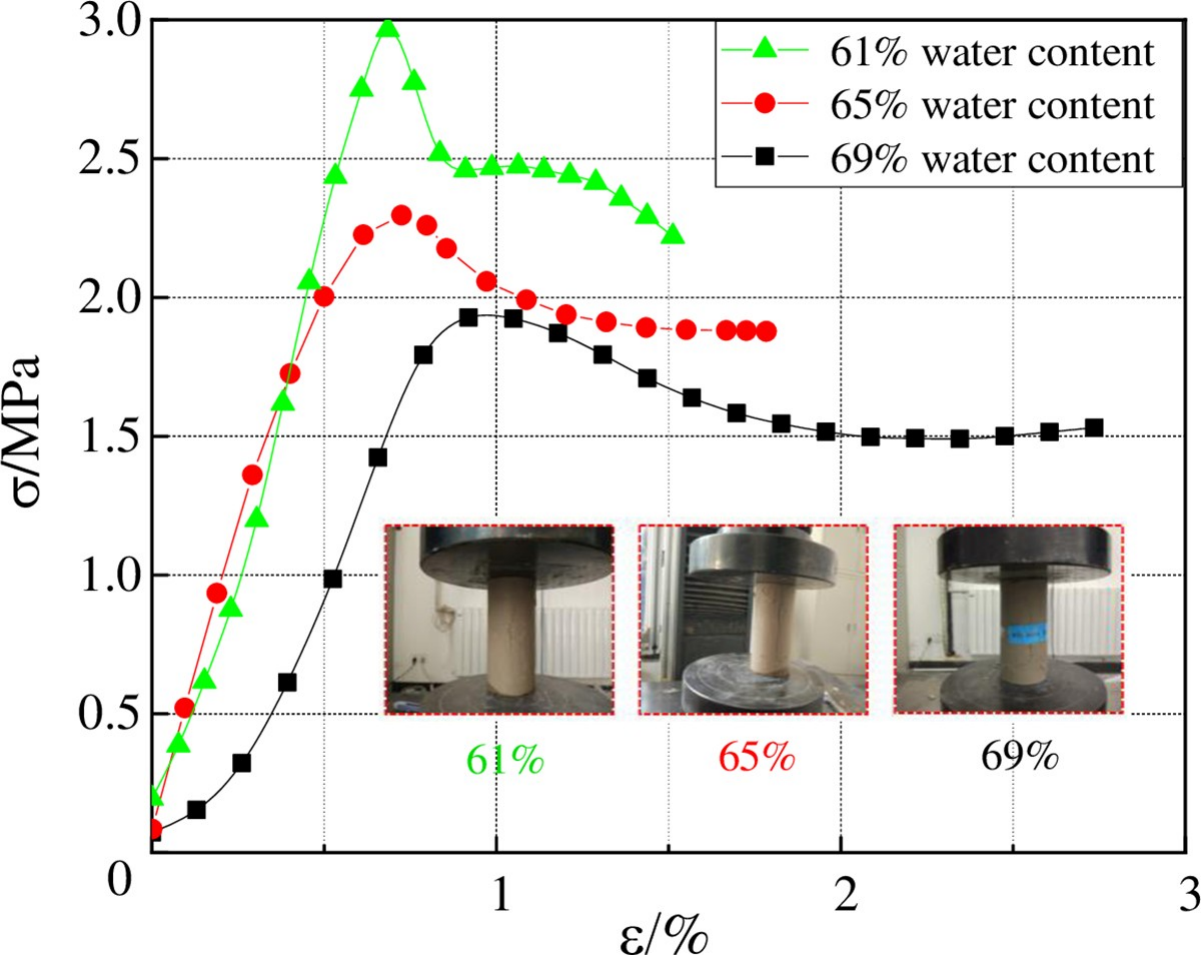
Uniaxial compressive stress-strain curve of high water filling materials.

Uniaxial bearing experiments were conducted on high water filling materials whose water content was 61%, 65%, and 69%, respectively. The samples measured 50 mm in diameter and 100 mm in height. For each group of moisture content samples, 5 samples are selected for the experiment, and the load is loaded at a constant speed at a rate of 0.2mm/min until the sample is broken. The average value of the 5 samples is taken as the test result. The corresponding stress-strain curves are shown in Fig 1 and the uniaxial compressive strength and various parameters are shown in Table 1. As can be seen from Fig 2, the bearing capacity of high water filling materials decreases as the water content increases; but after the peak, for the material with lower water content, its strength would fall remarkably and faster; for the material with higher water content, its stress changes more slowly after the peak value, appearing to be ductile failure. For the sample whose water content is 61%, its residual strength accounts for 45%~60% of the peak stress; for the sample whose water content is 65%, its residual strength accounts for 65%~75% of the peak stress; and for the sample whose water content reaches 69%, its residual strength makes up 75%~85% of the peak stress. Thus it can be known that ratio of residual strength of high-water materials to peak strength would increase as it water content increases.

**Table 1:** Uniaxial compressive strength and various parameters.

Water content	Peak stress *σ*_*c*_(MPa)	Elasticity modulus E_c_(10^2^MPa)	Secant modulus E_s_(10^2^MPa)	Peak strain ε_c_(%)
**61%**	3.1	5.5	3.6	0.75
**65%**	2.3	4.5	2.7	0.78
**69%**	1.9	3.1	1.9	0.98

### 2.3 Tri-axial compressive strength and macroscopic failure characteristics

**Fig 3:**
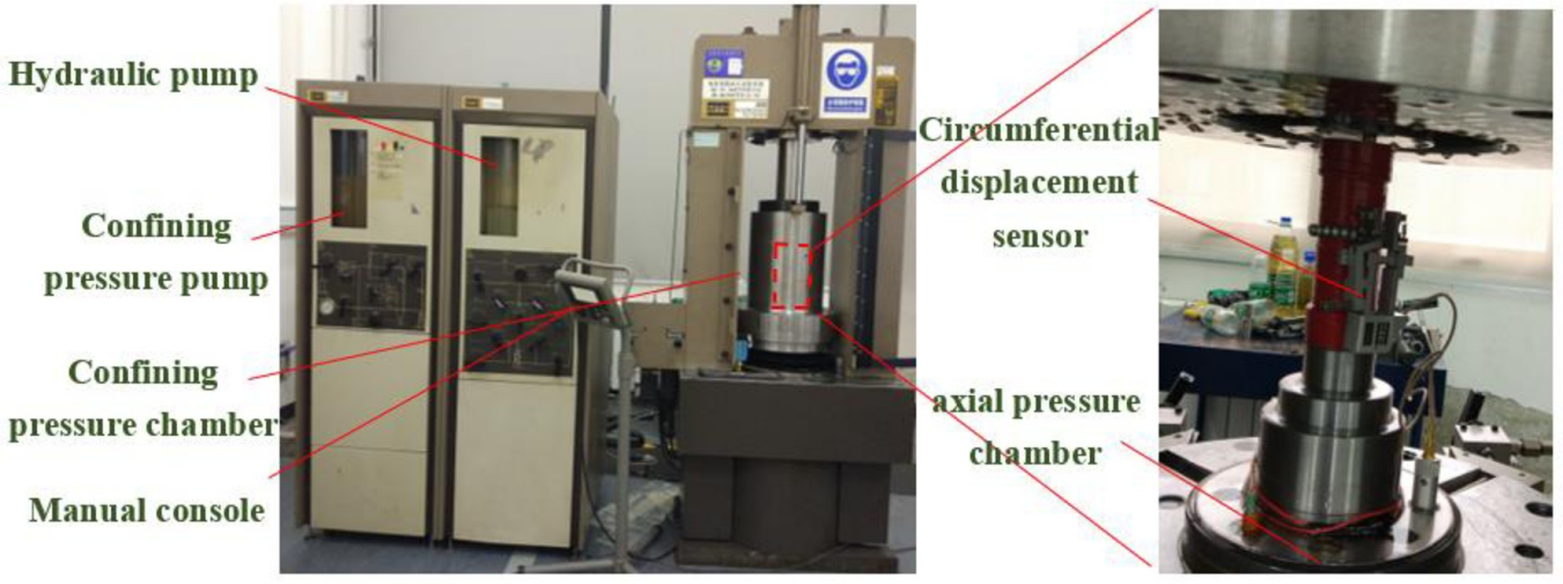
MTS 815 triaxial rock testing system.

**Fig 4:**
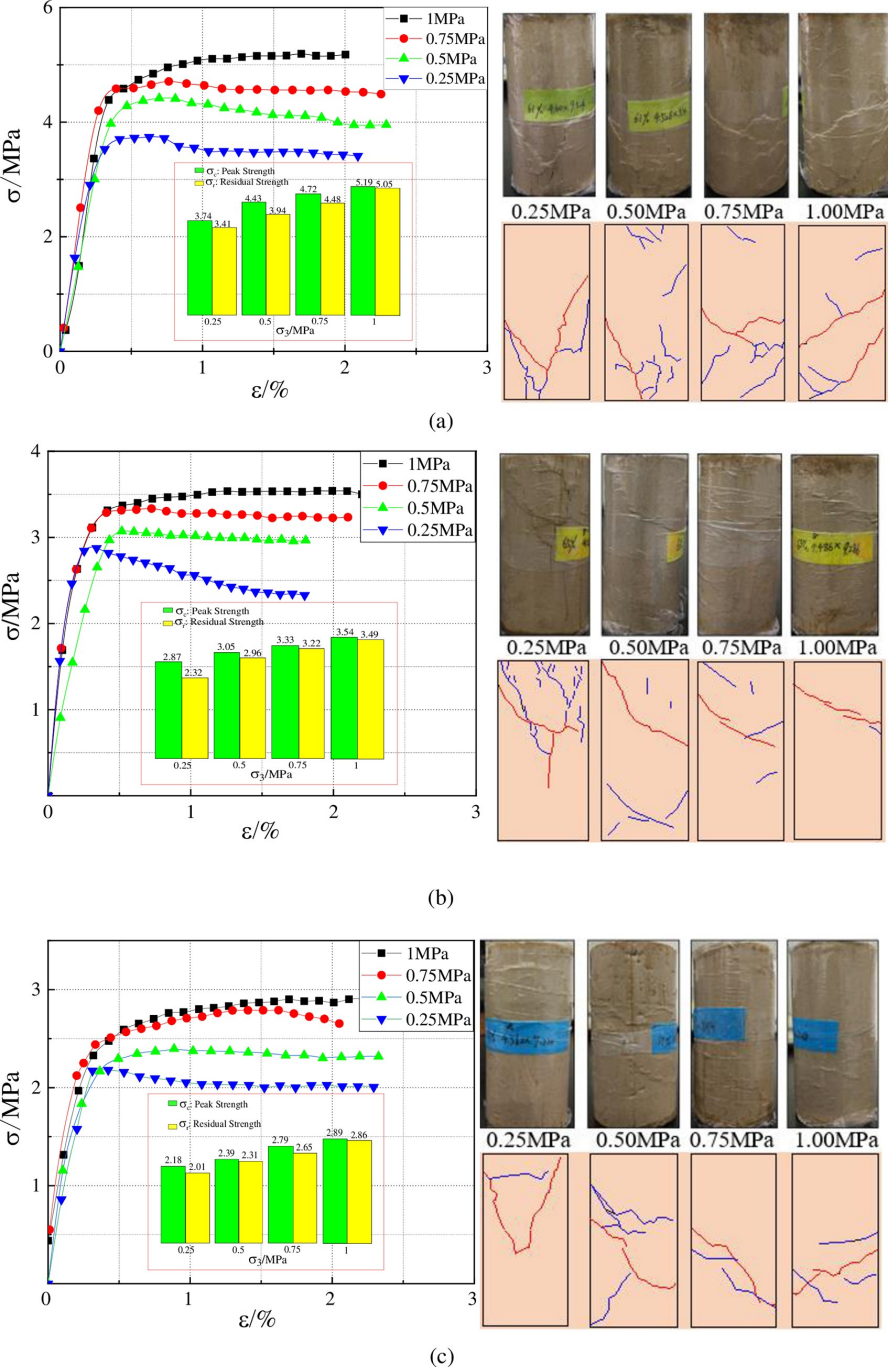
Stress-strain curves of materials with different water content under triaxial compression. (a) When the water content is 61%, (b) When the water content is 65%, (c) When the water content is 69%.

MTS815 electronic servo test machine was used to conduct tri-axial bearing experiments on high water filling materials with different water contents. The test machine is shown in Fig 3. The samples measured 50 mm in diameter and 100 mm in height. For each group of moisture content samples, 5 samples are selected for the experiment. The average value of the 5 samples is taken as the test result. The experiment scheme is to increase the confining pressure on high water filling materials with different water contents to 0.25MPa, 0.5MPa, 0.75MPa, and 1.0MPa in turn and the loading rate of confining pressure is 0.01MPa/s. Keep the confining pressure after loading to the set confining pressure. Then use the displacement control method to apply the axial load, and the loading rate is controlled to 0.01mm/s until the test block is broken. The results obtained are shown in Fig 4. As can be seen from the figure, when the confining pressure is relatively low, the stress-strain curve of high water filling materials is composed of four deformation stages: compaction stage, linear elastic stage, yield stage, and residual deformation stage after peak. The residual strength of high water filling materials varies in accordance with the water content. As the water content rises, the residual strength increases correspondingly, and the material tends to suffer from ductile failure. Under the effect of relatively higher confining pressure, the axial stress-strain curve tends to be a straight line after the peak value; the residual strength is virtually the same as the peak strength. The relatively high residual strength qualifies the high water filling materials to be applied in the underground filling environment.

The results of tri-axial compression experiments on high water filling materials with different water contents are shown in Table 2. As the confining pressure rises, the lateral deformation is confined more greatly in the loading process and the axial compressive strength increases remarkably. Under the same deformation condition, the compressive strength of the samples rises significantly, thus improving the elasticity modulus substantially. The Poisson’s ratio displays a downward trend as the water contents increases in that the higher the water content is, the more porous the sample is, and thus the higher its compression rate is under tri-axial pressure.

**Table 2:** Triaxial compressive strength and deformation parameters.

Water content	Confining pressure σ_3_ (MPa)	Peak stress σ_1c_ (MPa)	Peak strain ε_1c_ (%)	elasticity modulus E_1c_(GPa)	Poisson’s ratio μ
**61%**	0.25	3.70	0.70	1.23	0.24
	0.50	4.50	0.75	1.25	0.27
	0.75	4.70	0.75	1.48	0.18
	1.00	5.20	1.80	1.81	0.16
**65%**	0.25	2.90	0.45	0.81	0.22
	0.50	3.10	0.66	0.65	0.20
	0.75	3.36	0.65	0.83	0.17
	1.00	3.58	1.77	0.89	0.16
**69%**	0.25	2.20	0.36	0.65	0.21
	0.50	2.40	0.55	0.69	0.17
	0.75	2.81	1.60	0.69	0.14
	1.00	2.90	2.12	0.70	0.12

When the failure occurs under tri-axial compression, the mechanical performance of high water filling materials is no longer the mechanical properties of its own. Instead, the mechanical performance reflects the global structural effect caused by breaking the fracture surface through compression. Especially after the peak of stress-strain curve, besides the damage of internal structure, the rupture is compressed continuously inside the sample. So the failure mode of the sample is the comprehensive display of the whole failure process. Based on the tri-axial stress-strain curve and the form of the samples after failure, some representative samples are selected for analysis. Fig 4 displays the failure pictures of samples with different water contents under different confining pressure.

The red line represents the main crack and the blue line represents the secondary crack in Fig 4. Under the effect of uniaxial stress, the failure mode of samples is mainly multilayer split cracks which are parallel to the principal axis. And the cracks can mostly run through the whole sample. As the confining pressure rises, the failure mode of the samples begins to change. The angle between fracture surface and axial direction becomes larger gradually; the failure mode transforms from splitting failure to shear failure. Besides the principal cracks, a large number of minor cracks also appear around the principal ones on the surface. In addition to the influence of confining pressure on the development of cracks, it also depends on both lithology and mineral distribution. With the increase of confining pressure, the blocks of the sample after failure tend to be complete and the number of cracks on its surface decreases accordingly. This is because the failure tends to be ductile under the high confining pressure.

Besides the displacement in compaction stage and linear elastic stage, the deformation of high water filling material under tri-axial loading mainly comes from the slippage of fracture surface after the peak. The residual strength of samples is mainly determined by the strength of fracture surface. As the confining pressure increases, the angle of fracture surface is reduced significantly, which qualifies high water filling materials to bear greater stress and thus explains the phenomenon that the proportion of residual strength to peak strength increases with the rise of confining pressure.

### 2.4 Cohesion and friction angle

**Fig 5:**
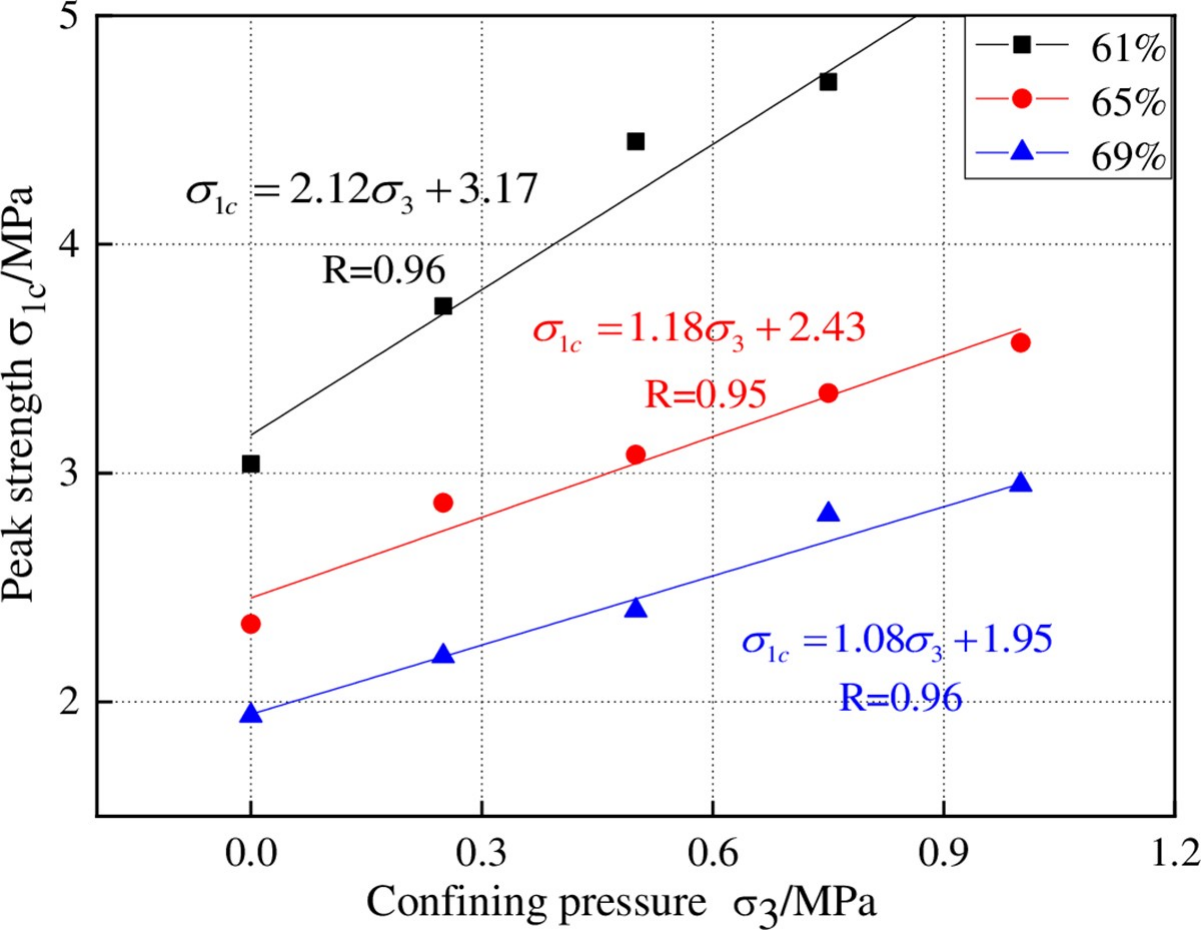
The curve of compressive strength versus confining pressure.

According to the tri-axial test data obtained, the curve of compressive strength (σ_1_) versus confining pressure (σ_3_) is drawn and shown in Fig 5. As can be known from the figure, the compressive strength of high water filling materials increases linearly as the confining pressure rises; the lower the water content is, the higher the compressive strength is. As the confining pressure increases gradually, the axial compressive strength displays an accelerated growth. For the sample whose water content is 69%, its uniaxial compressive strength is 1.93MPa. When the confining pressure rises, its compressive strength grows correspondingly. But when the confining pressure reaches 0.75MPa (σ_3_ = 0.75MPa), its compressive strength merely rises up to 2.8MPa; when the confining pressure reaches 1.0MPa (σ_3_ = 1.0MPa), the sample has already entered into a state of ductile failure. The peak value of its stress-strain curve has been vague, indicating that the sample has reached its bearing limit.

Since cohesion and friction angle are only related with the mechanical properties of the material, they remain unchanged even when the loading stress and confining pressure change. Therefore, the cohesion and friction angle can be calculated the linear fitting of confining pressure (σ_3_) and compressive strength (σ_1_). According to Mohr-Coulomb strength criterion, a linear relation exists between confining pressure (σ_3_) and compressive strength (σ_1_), which can be expressed as follows:

$$\sigma_{1}=K\sigma_{3}+C_{0}$$ (1)

where K and C_0_ are both parameters of strength criterion. Their relations with the cohesion (C) and friction angle (φ) of the sample can be expressed by the following equations:

$$K=\frac{1+\sin\varphi }{1-\sin\varphi }$$ (2)

$$C_{0}=\frac{2C\cos\varphi }{1‐\sin\varphi }$$ (3)

hence, the value of cohesion and friction angle of the sample can be calculated through the following equations:

$$\varphi =\arcsin\frac{K-1}{K+1}$$ (4)

$$C=C_{0}\frac{1-\sin\varphi }{2\;\cos\varphi }$$ (5)

The calculation results of cohesion and friction angle are shown in Table 3. The calculation results of residual cohesion and residual friction angle are shown in Table 4. As can be seen, the cohesion does not change remarkably when the water content rises; instead, it is the friction angle of the material that exerts greater impact on the compressive strength. As the water content increases, the pores inside the sample tend to be larger and more and its internal structure becomes loose, causing the sharp reduction of friction and occlusal force among particles inside the sample. The failure mode is mainly shear failure. It can be concluded that the residual cohesion of the high-water material is reduced relative to the cohesion before the peak stress, but the residual internal friction angle is increased relative to the internal friction angle before the peak stress. This has the same properties as rocks. However, as the moisture content of the high-water material increases, there is more pore water inside, which ensures that the loss of cohesion is small, and the residual strength of the high-water material accounts for a larger proportion of the peak strength.

**Table 3:** Relations between cohesion, friction angle and water content.

Water content	61%	65%	69%
**cohesion C/MPa**	1.09	1.12	0.94
**friction angle φ/°**	21.04	4.74	2.21

**Table 4:** Relations between residual cohesion, residual friction angle and water content.

Water content	61%	65%	69%
**cohesion C**_**r**_**/MPa**	0.70	0.69	0.67
**friction angle φ**_**r**_**/°**	28.81	16.42	9.27

## 3 The residual strength characteristics of high water filling materials

### 3.1 The characteristics of residual strength variation

After the peak of stress-strain curve, as the strain increases continuously, the stress tends to be stable gradually and reaches some certain value ultimately which is the residual strength of the material. As the elastic deformation of the sample develops after the peak, the internal structure deteriorates constantly until failure occurs. The fissures inside the sample develop further and perforate into a macroscopic fracture surface in the end. With constant loading, block sliding occurs on the fracture surface. The yield surface in the residual state is different from the yield rupture surface at the peak. Accordingly, the strength coefficient, cohesion (C_r_), and friction angle (φ_r_) are all different from those at the peak.

**Fig 6:**
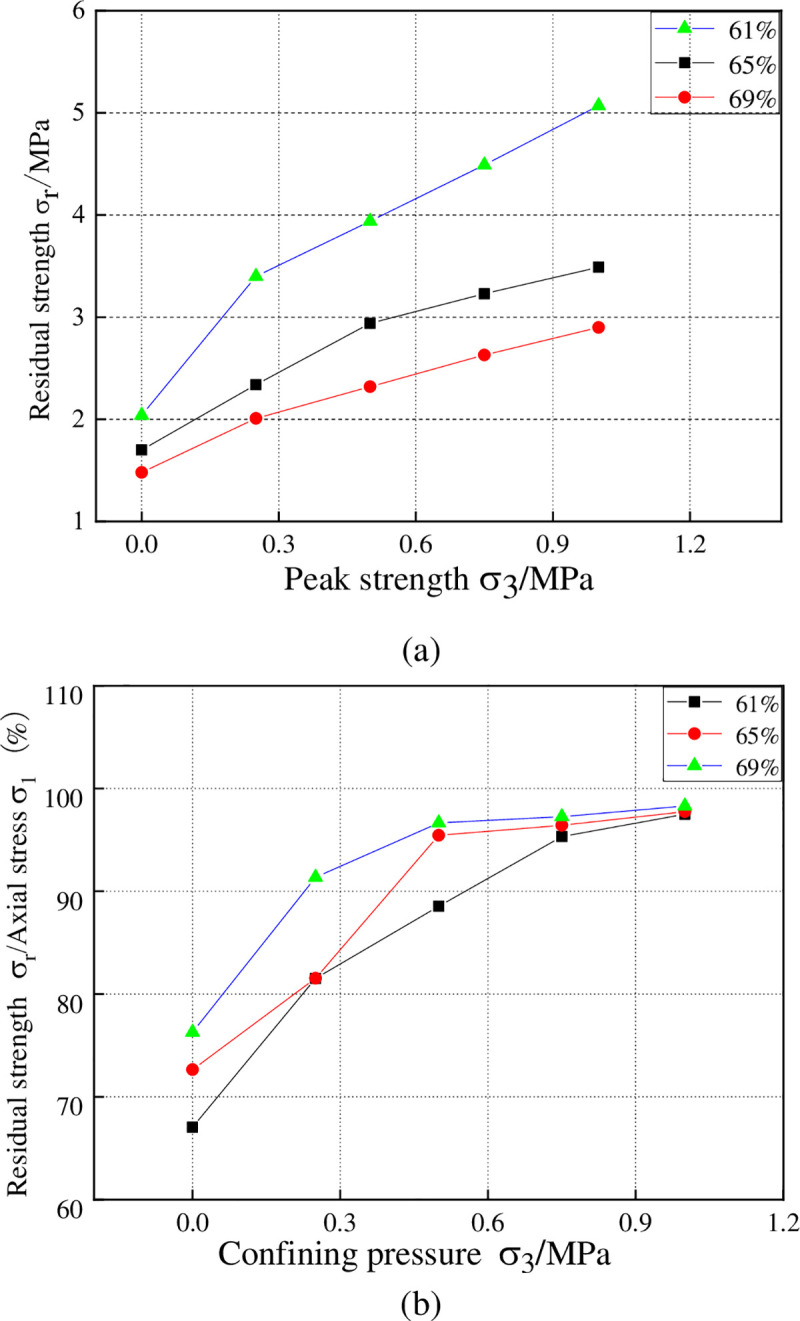
The change curve of residual strength under different water contents. (a)The change curve of residual strength versus confining pressure, (b) The change curve of residual strength proportion.

As is shown in Fig 6(A), the residual strength rises as the confining pressure increases, which conforms to the variation trend of axial compressive strength. But from Fig 6(B) it can be known that the proportion of residual strength to peak strength would rise as the confining pressure grows, which justifies why the change curve of residual strength does not follow a linear growth pattern strictly. As the figure shows, the residual strength would decline as the water content rises; but the proportion of residual strength to peak strength would rise as the water content rises, which is in line with the change rule of uniaxial residual strength. This is because high water content means that more pore water exists in the failure process of the peak, which in turn guarantees the relatively small loss of cohesion. In this case, the integrity of the sample is better preserved and the sample displays the characteristics of ductile deformation.

### 3.2 The improvement of residual strength characterization model

Currently, the parameters of residual strength are usually determined by Mohr-Coulomb model, Joseph model or H-B model fitting. This paper conducts fitting process on the residual strength of the sample based on the existing model. Then it is put forward to use the degree of cohesion loss to characterize the integrity of the sample on the basis of H-B model. An improved H-B model is established which is suitable for the residual strength characterization. In the broad sense, H-B criterion is a method to determine rock strength by means of the actual geological conditions. Based on a large quantity of test data, Hoek and Brown put forward H-B failure criterion by way of data fitting, which is expressed by the following equation.


$$\sigma_{1}=\sigma_{3}+\sigma_{c}{\left(m_{i}\frac{\sigma_{3}}{\sigma_{c}}+s\right)}^{a}$$ (6)

Where *σ*_1_ and *σ*_3_ denote the axial compressive strength and confining pressure of the material under tri-axial stress; numerically, *σ*_*c*_ can be taken as the model parameter of uniaxial compressive strength; *m*_*i*_ stands for model parameter and s value is similar to the cohesion parameter of the material which is determined by the integrity of the sample. To put it simple, if the rock mass is complete, s = 1; the more broken the rock is, the closer its value of s is to zero; a denotes parameter model, which can improve the fitting degree and qualify H-B failure criterion for fractured rock mass.

The original H-B failure criterion can be used to describe the stress curve of many materials, but its variables are relatively complicated. As is stated above, according to H-B failure criterion, *m*_*i*_ is the equivalent of friction angle of rock and s is the equivalent of cohesion of rock [24]. So this paper uses *φ*_*r*_ to substitute *m*_*i*_ and the degree of cohesion loss to characterize the value of s. Combined with M-C model, the known cohesion is substituted into Eq (6).

$$m_{i}=\varphi_{r}$$ (7)

$$s=C_{r}/C$$ (8)

$$\sigma_{c}=\sigma_{cr}$$ (9)

where *φ*_*r*_ denotes the residual friction angle obtained through M-C model; C, and Cr are the cohesion of the sample and the residual cohesion; and *σ*_*cr*_ denotes the model parameter of residual strength.

After improvement, the relationship between residual strength and confining pressure can be expressed by the following equation:

$$\sigma_{1r}=\sigma_{3}+\sigma_{cr}{\left(\varphi_{r}\frac{\sigma_{3}}{\sigma_{cr}}+\frac{C_{r}}{C}\right)}^{a}$$ (10)

where there are only two variables: *φ*_*cr*_ and a. Numerically, *φ*_*cr*_ can be taken as the residual strength of uniaxial compression. The improved H-B failure criterion model endows the variables with physical significance. When the general H-B criterion is used to fit the sample, the value of s is set as 1 if the rock mass is complete; the value of s would be set as 0 if the rock mass is not complete. But this is not suitable for material with ductile failures. The improved H-B model uses the ratio of residual cohesion to cohesion to characterize the value of s, thus reflecting the change rule of residual strength of ductile materials more competently. When the effect of water is taken into consideration, effective principal stress should be used instead of principal stress. A large number of experimental studies have found that the influence of pore water pressure is small, so the influence of pore water pressure is not considered.

**Fig 7:**
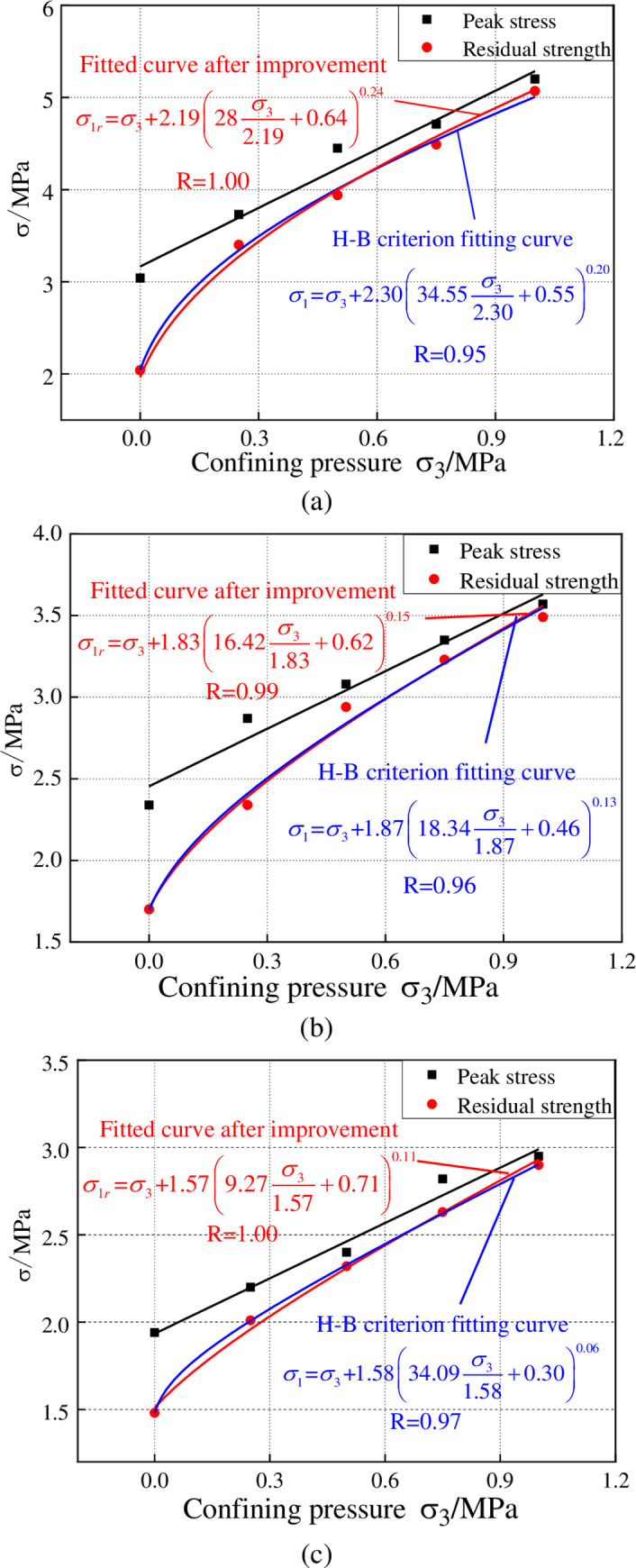
The fitting chart of residual strength based on H-B criterion and the improved model. (a) When the water content is 61%, (b) When the water content is 65%, (c) When the water content is 69%.

As can be known from Fig 7, after the introduction of cohesion and friction angle, H-B criterion can fit the residual strength curve of high water filling materials better. The fitting coefficient of the samples with three water contents is 1.00, 0.99, 1.00, respectively, all higher than the unassigned H-B criterion fitting curves. This indicates that the improved H-B criterion can reflect the change rule of the residual strength versus confining pressure.

## 4 Conclusion

Based on uniaxial and tri-axial compression experiments, this paper studies the characteristics of the stress-strain curve, bearing capacity, and residual strength of high water filling material. The conclusions drawn are as follows:

1. Under uniaxial loading, the bearing capacity of high water filling materials would decline as the water content increases. The lower the water content is, the more significantly and faster the strength falls after the peak. The failure in this case tends to be brittle failure. Under tri-axial loading, when the confining pressure is relatively lower, the residual strength displays a clear upward trend. The higher the water content is, the more remarkably the residual strength rises; when the confining pressure is relatively higher, the axial stress-strain curve is close to a straight line after the peak value and there is no significant variation between the residual strength and peak strength. The relatively high residual strength makes high water filling materials more suitable for the underground filling environment.
2. According to Mohr-Coulomb strength criterion, as the water content rises, the cohesion of the high water filling materials does not change much. Instead, the friction angle of the material exerts greater impact on its compressive strength. When the water content rises, the pores inside the samples become larger and more, making its internal structure become loose. In this case, the surface friction and occlusal force between particles inside the sample reduce drastically; the failure mode is mainly shear failure.
3. The residual strength is firstly fitted with the original model. Then on the basis of H-B model, friction angle *φ*_*r*_ is introduced to substitute model parameter *m*_*i*_, and the degree of cohesion loss is used to characterize the value of s. So the improved H-B model is established to characterize the residual strength of materials with ductile failure characteristics.
4. Under tri-axial loading, the angle between fracture surface and axial direction would increase as the confining pressure rises. The failure of the sample transforms from splitting failure to shear failure. In addition to principal cracks, a large number of minor cracks appear around the principal ones on the surface of the samples. Besides the displacement in elastic compaction stage, the deformation of high water filling material under tri-axial loading is mainly caused by the slippage of fracture surface after the peak. The residual strength of samples is largely determined by the strength of fracture surface.

## Supporting information

S1 Data (RAR)
